# Microbiome confounders and quantitative profiling challenge predicted microbial targets in colorectal cancer development

**DOI:** 10.1038/s41591-024-02963-2

**Published:** 2024-04-30

**Authors:** Raúl Y. Tito, Sara Verbandt, Marta Aguirre Vazquez, Leo Lahti, Chloe Verspecht, Verónica Lloréns-Rico, Sara Vieira-Silva, Janine Arts, Gwen Falony, Evelien Dekker, Joke Reumers, Sabine Tejpar, Jeroen Raes

**Affiliations:** 1 Laboratory of Molecular Bacteriology, Department of Microbiology and Immunology, Rega Institute, Katholieke Universiteit Leuven, Leuven, Belgium; 2 Center for Microbiology, Vlaams Instituut voor Biotechnologie, Leuven, Belgium; 3 Digestive Oncology, Department of Oncology, Katholieke Universiteit Leuven, Leuven, Belgium; 4 Department of Computing, University of Turku, Turku, Finland; 5 Systems Biology of Host–Microbiome Interactions Laboratory, Principe Felipe Research Center (CIPF), Valencia, Spain; 6 Institute of Medical Microbiology and Hygiene and Research Center for Immunotherapy, University Medical Center of the Johannes Gutenberg-University Mainz, Mainz, Germany; 7 Institute of Molecular Biology, Mainz, Germany; 8 Oncology, Janssen Pharmaceutica NV, Beerse, Belgium; 9 Department of Gastroenterology and Hepatology, Amsterdam University Medical Centers, Amsterdam, the Netherlands; 10 Therapeutics Discovery, Janssen Pharmaceutica NV, Beerse, Belgium

**Keywords:** Diagnostic markers, Microbiome, Colon cancer

## Abstract

Despite substantial progress in cancer microbiome research, recognized confounders and advances in absolute microbiome quantification remain underused; this raises concerns regarding potential spurious associations. Here we study the fecal microbiota of 589 patients at different colorectal cancer (CRC) stages and compare observations with up to 15 published studies (4,439 patients and controls total). Using quantitative microbiome profiling based on 16S ribosomal RNA amplicon sequencing, combined with rigorous confounder control, we identified transit time, fecal calprotectin (intestinal inflammation) and body mass index as primary microbial covariates, superseding variance explained by CRC diagnostic groups. Well-established microbiome CRC targets, such as *Fusobacterium nucleatum*, did not significantly associate with CRC diagnostic groups (healthy, adenoma and carcinoma) when controlling for these covariates. In contrast, the associations of *Anaerococcus vaginalis*, *Dialister pneumosintes*, *Parvimonas micra*, *Peptostreptococcus anaerobius*, *Porphyromonas asaccharolytica* and *Prevotella intermedia* remained robust, highlighting their future target potential. Finally, control individuals (age 22–80 years, mean 57.7 years, standard deviation 11.3) meeting criteria for colonoscopy (for example, through a positive fecal immunochemical test) but without colonic lesions are enriched for the dysbiotic Bacteroides2 enterotype, emphasizing uncertainties in defining healthy controls in cancer microbiome research. Together, these results indicate the importance of quantitative microbiome profiling and covariate control for biomarker identification in CRC microbiome studies.

## Abstract (web summary)

Controlling for confounders calls into question the robustness of some associations of microbiota with colorectal cancer stages.

## Main

Colorectal cancer (CRC) incidence is steadily increasing^1^, especially in people under 50 years^2^. It is estimated that approximately 16 and approximately 14 individuals per 100,000 people in the United States and Belgium, respectively, die every year from CRC^3^. As medical interventions can effectively reduce CRC progression and associated mortality, it is imperative to identify individuals at increased risk.

Colonoscopies with polypectomy of adenomas reduce up to 90% of CRC risk^4^. Early identification of individuals with polyps would reduce the global burden of CRC. Yet, ascertainment of patients at an increased risk remains challenging, highlighting the need for population-wide screening.

Microbiota shifts have been associated with a wide array of disease phenotypes^5^. Some bacterial markers, such as *Fusobacterium*, have been reported enriched in lesions and stools of patients with CRC^6–14^ across developing and developed countries^15^, suggesting a potential role for microbiome-based diagnostics and/or prognostics.

Although microbiome profiles are affected by multiple variables that may confound or compound biological phenomena, covariate control is far from standard. For example, moisture content, a proxy for transit time, remains uncontrolled despite showing the biggest explanatory power for overall gut microbiota variation in multiple cohorts^16,17^. Intestinal inflammation, measured as fecal calprotectin^18,19^ that reflects increased neutrophil shedding into the intestinal lumen^20^, is more sensitive than fecal occult blood for identifying patients with CRC^21^, thus a potential untapped target for molecular stool CRC-screening^19^.

Relative microbiome profiling (RMP, taxon abundances are expressed in percentages) remains the dominant approach in microbiome research. However, given issues with compositionality^22^ and interpretation of relative profiles^23^, the use of experimental and quantitative approaches is increasingly recommended^23–25^. This reduces both false-positive and false-negative rates in downstream analyses, thereby lowering the risk of erroneous interpretation of microbiome associations, and allows focusing clinical programs on biologically relevant targets^25^. Although quantitative microbiome profiling (QMP) facilitates normalized comparisons across different samples or conditions^24,25^, so far, no QMP CRC microbiota studies were performed.

In this Article, we address these two gaps in CRC microbiota studies: (1) to quantitively characterize the microbiota profile associated with malignant colonic transformation and (2) to identify microbiota covariates that may obscure biological phenomena behind microbiota-CRC associations. To this end, we examined the microbial profiles of 589 Belgian patients from Universitair Ziekenhuis Leuven (UZL) who warranted colonoscopies based on clinical presentations, including patients with CRC, and compared these to existing published datasets (total *n* = 4,439 patients and controls). To the best of our knowledge, this is the first large scale study of the gut microbiota across colonic cancer developmental stages that combines QMP analysis with extensive analysis of microbiota covariates to disentangle disease-associated from confounder-based signals to identify taxa specifically associated with CRC.

## Results

### Intestinal inflammation is higher in patients with colorectal tumors

**Fig. 1:**
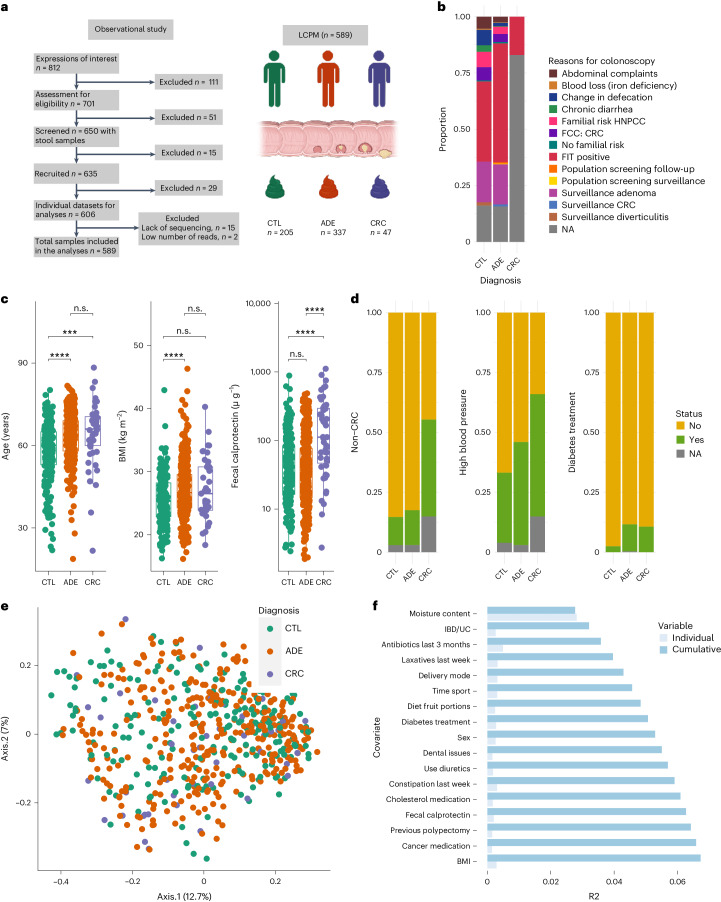
The LCPM cohort and gut microbiota covariates in CRC progression. **a**, STROBE flowchart and cohort size. CTL represents patients without colonic lesions, ADE denotes patients with colonic polyps and CRC refers to patients with colorectal tumors (generated in BioRender.com). **b**, Colonoscopy referral reasons for patients of the LCPM cohort: positive FIT, adenoma surveillance, familial risk cancer (FCC), hereditary nonpolyposis CRC (HNPCC) and changes in defecation. NA, denotes the proportion of patients without information. **c**, Age, BMI and calprotectin are associated with diagnosis groups. The patients without lesions were younger (*n* = 589, two-sided KW test *χ*^2^ = 35.77, adjusted *P* = 2.6 × 10^−7^; phD tests) and had lower BMI (*n* = 553, two-sided KW test *χ*^2^ = 15.73, adjusted *P* = 1.9 × 10^−3^; phD tests), while patients with tumors had higher fecal calprotectin levels (*n* = 583, two-sided KW test *χ*^2^ = 29.43, adjusted *P* = 3.0 × 10^−6^; phD tests, adjusted ***P <0.001, **P <0.01, *P <0.05 and n.s., non-significant *P* > 0.05; Supplementary Table 3). The box plot center represents the median value whiskers extend from the quartiles to the last data point within 1.5 times of the interquartile range, with outliers beyond. **d**, Previous non-CRC cancer, high blood pressure and diabetes treatment are associated with the distribution of diagnosis groups. The patients with CRC have a higher proportion of previous cancer (47.5% versus 15.0 % and 12.1%, two-sided CS test, CV effect size of 0.24, *χ*^2^ = 31.65, d.f. of 2, adjusted *P* = 1.98 × 10^−2^) and high blood pressure (60.0% versus 44.3% and 30.5%, CV of 0.17, two-sided CS test, *χ*^2^ = 16.55, d.f. of 2, adjusted *P* = 1.98 × 10^−2^) while the CTL group has the lowest proportion of patients with diabetes treatment (2.4% versus 10.3 and 10.6, two-sided CV effect size of 0.15, CS test, *χ*^2^ = 13.79, d.f. of 2, adjusted *P* = 1.98 × 10^−2^). **e**, PCoA on BCD representing QMP species-level microbiota variation in the LCPM cohort (*n* = 589), PCoA1 (Axis.1) and PCoA2 (Axis.2) respectively explained 12.7% and 7% of the variance. Each dot represents one sample, colored by assigned diagnosis group. **f**, Cumulative effect sizes of significant covariates on microbiota community variation (cumulative bars; stepwise dbRDA on BCD) as compared to individual effect sizes (R^2^) assuming covariate independence in the LCPM cohort (*n* = 589; Supplementary Table 5). UC, ulcerative colitis. Source data

We recruited 650 volunteers referred for colonoscopy and colonic resections at UZL between 2017 and 2018 who provided a stool sample before the colonic procedure. Most participants were from the Flemish region of Belgium. For this study, cancer developmental stages were defined as diagnosis groups, and we classified participants into three groups according to a thorough colonoscopy and clinical assessment: (1) patients without evidence of colonic lesions (CTLs, *n* = 205), (2) patients with polyps (considering polyps as a precancerous lesion; *n* < 10 and size between 6 and 10 mm) (ADE, *n* = 337) and (3) patients with CRC (*n* = 47; 2 (4%) stage 0, 14 (30%) stage I, 13 (28%) stage II, 11 (23%) stage III, 3 (6%) stage IV and 4 (9%) of undetermined stage). We excluded patients outside these criteria, as well as those with insufficient clinical and molecular data. The final Leuven CRC Progression Microbiome (LCPM) study cohort consisted of 589 patients. The most frequent indications for colonoscopy were either a positive fecal immunochemical test (FIT) or adenoma surveillance. Other indications included familial risk, abdominal symptoms and change in bowel habits (Fig. 1a and Supplementary Table 1). The study was registered at clinicaltrials.gov (NCT02947607).

We collected an extensive set of 165 universal metadata variables (nonspecific for any of the three groups) from each participant. After curation, we excluded variables that were colinear (if Pearson |*r*| > 0.8, we kept the variable with fewer missing data) or had incomplete data collection (variables missing more than 20% of the values). The final set consisted of 95 high-quality variables (Supplementary Table 2).

To identify metadata variables associated with diagnosis groups, we applied two statistical approaches: (1) nonparametric Kruskal–Wallis (KW) test and its *η*^2^ effect size (Supplementary Table 3) for all numerical variables and (2) chi-square (CS) tests and Cramer’s V effect size (CV) (Supplementary Table 4) for categorical variables, followed by the Benjamini–Hochberg method for multiple testing correction (adjusted *P*). We found eight variables associated with diagnosis groups (false discovery rate <5%), namely: age, body mass index (BMI), calprotectin, reported hours of sleep, previous cancer (including CRC), dental status (complete, partial and so on), diabetes treatment and high blood pressure (Supplementary Tables 3 and 4). The CTL patients were younger (*n* = 589, KW test, *η*^2^ = 0.058, *χ*^2^ = 35.77, adjusted *P* = 2.6 × 10^−7^; post hoc Dunn (phD) tests, adjusted *P* < 0.05 for CTL versus ADE or CRC groups), had a lower BMI (*n* = 553, KW test, *η*^2^ = 0.023, *χ*^2^ = 15.73, adjusted *P* = 1.9 × 10^−3^; phD tests, adjusted *P* < 0.05 for CTL versus ADE) and reported fewer hours of sleep than participants from the other two diagnosis groups (*n* = 557, KW test, *η*^2^ = 0.019, *χ*^2^ = 13.41, adjusted *P* = 4.6 × 10^−3^; phD tests, adjusted *P* < 0.05 for CTL versus ADE; Fig. 1; see Supplementary Table 3 for full results). Moisture content, an important microbiota covariate^16^, was not significant across diagnosis groups (*n* = 589, KW test, *η*^2^ = −0.001, *χ*^2^ = 1.32, adjusted *P* = 7.0 × 10^−1^).

The calprotectin levels were positively associated with malignant transformation. The patients with CRC showed higher intestinal inflammation, measured by fecal calprotectin^18,26^ (Fig. 1a and Supplementary Table 3). Specifically, CRC exhibited higher levels (219.42 µg g^−1^, range 2.74–1,114.42, *n* = 47) compared to ADE (70.24 µg g^−1^, range 1.87–487.21, *n* = 337) or CTL (73.25 µg g^−1^, range 2.42–884.82, *n* = 202) (Fig. 1a, *N* = 583, KW test, *η*^2^ = 0.047, *χ*^2^ = 29.43, adjusted *P* = 3.0 × 10^−6^; phD tests, adjusted *P* < 0.05 for CRC versus CTL and CRC versus ADE). We also observed increased fecal calprotectin in patients reporting previous cancers (primarily breast and prostate cancer) (Wilcoxon ranksum (WR) test, *W* = 11,067, adjusted *P* = 4.1 × 10^−3^), consumption of cancer medication (WR test, *W* = 3,671, adjusted *P* < 0.05), heartburn complaints (WR test, *W* = 11,067, adjusted *P* = 1.0 × 10^−10^) and lower dietary fiber (WR test, *W* = 20,964, adjusted *P* = 3.3 × 10^−2^).

The history of chronic diseases was distinct across diagnosis groups. The patients with CRC showed higher proportions of previous non-CRC cancer (47.5% versus 15.0 % and 12.1%, CS test, CV of 0.24, *χ*^2^ = 31.65, d.f. of 2, adjusted *P* = 1.98 × 10^−2^) and high blood pressure (60.0% versus 44.3% and 30.5%, CS test, CV of 0.17, *χ*^2^ = 16.55, d.f. of 2, adjusted *P* = 1.98 × 10^−2^) (Fig. 1b and Supplementary Table 4). The CTL group had the lowest diabetes treatment (2.4% versus 10.3% and 10.6%, CS test, CV of 0.15, *χ*^2^ = 13.79, d.f. of 2, adjusted *P* = 1.98 × 10^−2^) (Fig. 1b and Supplementary Table 4) and mostly complete dental sets (53.3% versus 35.2% and 32.5%, CS test, CV of 0.03, *χ*^2^ = 30.78, d.f. of 10, adjusted *P* = 1.98 × 10^−2^) (Supplementary Table 4).

### Known confounders, not diagnosis groups, explain overall microbiota variation across CRC developmental stages

The influence of microbiota covariates and the quantitative amplitude of observed microbiota shifts are understudied in CRC. We combined sequencing data with flow cytometry measurements of fecal microbial load^23^ to generate QMP data from our study cohort.^23^ We studied the QMP variation in the context of the 94 potential covariates mentioned above (the 95th being microbial load) using established procedures^17^.

A principal coordinate analysis (PCoA; Fig. 1c) on a species-level Bray–Curtis dissimilarity (BCD) matrix revealed no significant separation between diagnosis groups. Furthermore, no difference in total microbial load was found between groups (*n* = 589, KW test, *χ*^2^ = 0.68, adjusted *P* = 8.2 × 10^−1^). Distance-based redundancy analysis (dbRDA) revealed 24 microbiota covariates associated with microbial variation in this cohort (Fig. 1d and Supplementary Table 5). We identified 17 nonredundant covariates that jointly explained 6.7% of microbiota compositional variation (Supplementary Table 5).

Consistent with previous reports^16,17^, moisture content exhibited the highest explanatory value (2.8%) of all covariates (*n* = 589, stepwise dbRDA, *R*^2^ = 2.8%, adjusted *P* *=* 2 × 10^−3^). Intestinal bowel disease/ulcerative colitis (IBD/UC) status, a CRC-risk factor, possibly associated with its microbial dysbiotic community and intestinal inflammation^27^, was the second largest covariate. IBD/UC explained 0.4% of the microbiota variation (*n* = 569, stepwise dbRDA, *R*^2^ = 0.4%, adjusted *P* = 2 × 10^−3^). Other top microbiota covariates included antibiotics and laxatives use (Fig. 1d). Delivery mode (cesarean or natural birth) explained 0.3% variation (*n* = 533, stepwise dbRDA, *R*^2^ = 0.3%, adjusted *P* = 2 ×10^−3^), although it is probably confounded by diet in this cohort (proportion of dietary vegetables; CS test, *χ*^2^ = 33.09, d.f. of 14, *P* = 2.8 × 10^−3^, adjusted *P* < 0.05). Intestinal inflammation (fecal calprotectin) explained 0.2% (*n* = 583, stepwise dbRDA, *R*^2^ = 0.2%, adjusted *P* = 2.6 × 10^−2^). In contrast with our previous study in the Flemish population (Flemish Gut Flora Project, FGFP)^17^, age did not explain microbiota variation (*n* = 589, univariate dbRDA, *R*^2^ = 0.2%, adjusted *P* = 5.9 × 10^−2^). Surprisingly, the cancer diagnosis group (CTL, ADE and CRC), as a covariate, was not associated with microbial variation (*n* = 589, univariate dbRDA, *R*^2^ = 0.2%, adjusted *P* = 0.22; Supplementary Table 5).

### *Fusobacterium* association with CRC stages disappears when controlling for confounders or when using QMP

**Fig. 2:**
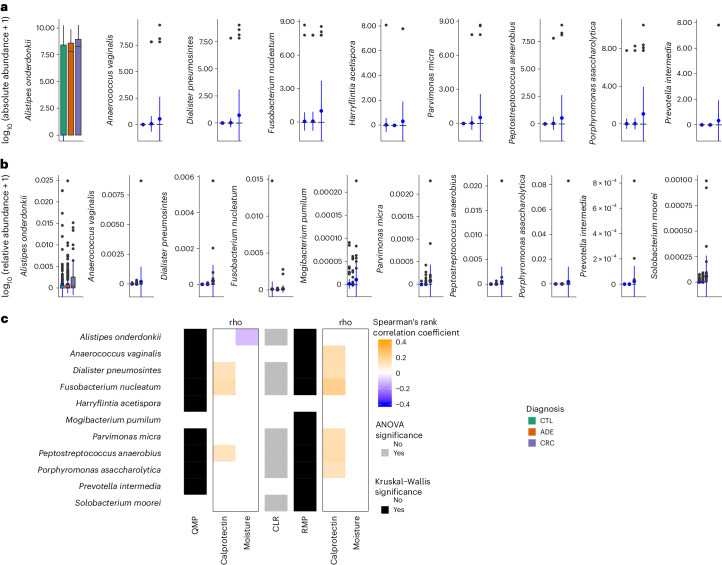
Microbial biomarkers in CRC progression. **a**, Nine species were identified with differential absolute abundance across diagnosis groups (*n* = 589, KW test, adjusted *P* < 0.05; Supplementary Table 7). **b**, Ten species were identified with differential relative abundance across diagnosis groups (*n* = 589, KW test, adjusted *P* < 0.05; Supplementary Table 7). The center of the box plot represents the median value of the data, and the whiskers extend from the quartiles to the last data point within 1.5 times of the interquartile range, with outliers beyond. The blue circles represent the mean. **c**, Biomarkers associations and their confounders. Species Spearman’s rank correlation with calprotectin levels and moisture proportions using QMP (first rho column panel) and RMP (second rho column panel) data. The effect size of the associations between species and calprotectin, moisture and diagnosis variables for QMP and RMP (*n* = 589, Spearman’s rank correlation comparison, adjusted *P* < 0.05). Significant associations were tested using two-sided KW tests for QMP and RMP data and ANOVA for CLR data. The associations for *Harryflintia acetispora*, *Parvimonas micra* and *Prevotella intermedia* are sensitive to bias by the extreme values (absolute abundance) in the higher range. Removing these values leads to loss of significance. As rank-based approaches were used, it is not clear if this loss is due to the strength of the signal or the loss of power. Source data

**Extended Data Fig. 1:**
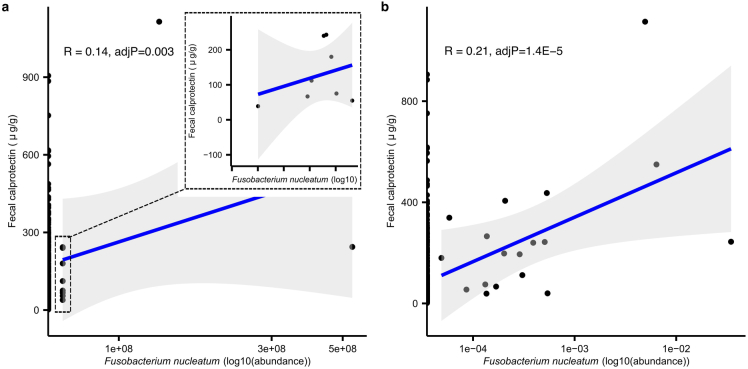
Association of intestinal inflammation with *Fusobacterium nucleatum*. Intestinal calprotectin levels associate Fusobacterium nucleatum absolute (**a)** and relative (**b**) abundance in the LCMP. Two-sided Spearman rank correlation (adjP <0.05) and ‘x’ axes are log 10 transformed just for plotting. To rule out that the observed association is driven by a few samples with high abundance of Fusobacterium nucleatum, panel a has an insert of the plot removing samples with Fusobacterium nucleatum values above 1E8 cells per gram of stool. Best-fitting regression line in blue and 95% confidence interval shown in grey shading.

**Extended Data Fig. 2:**
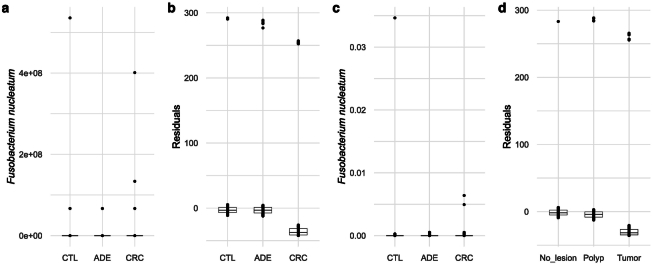
*Fusobacterium nucleatum* abundances before and after correction for intestinal calprotectin across diagnosis groups. Absolute abundance of Fusobacterium nucleatum before (**a**) and after (**b**) correcting for intestinal calprotectin. Relative abundance of Fusobacterium nucleatum before (**c**) and after (**d**) correcting for intestinal calprotectin. The whiskers extend from the quartiles to the last data point within 1.5× of the interquartile range, with outliers beyond. The ‘y’ axes for (a) are log 10 transformed values (absolute abundance +1). The whiskers extend from the quartiles to the last data point within 1.5× of the interquartile range, with outliers beyond.

Microbiota signals can be specific to taxonomic groups and, thus, not reflected in broad community shifts. While a multitude of microbial associations have been reported in CRC studies using RMP^6–8,13^, we used QMP to identify species whose absolute abundance associated with diagnosis groups. The comparisons were limited to the 138 species with a prevalence of greater than 5% in at least one of the diagnosis groups of the LCPM cohort (Supplementary Table 6). Only eight species showed significant differential abundance (absolute or relative) among diagnosis groups: *Anaerococcus vaginalis* (*Anaerococcus obesiensis*), *Alistipes onderdonkii*, *Dialister pneumosintes*, *Fusobacterium nucleatum*, *Parvimonas micra*, *Peptostreptococcus anaerobius*, *Porphyromonas asaccharolytica* and *Prevotella intermedia* (KW test, adjusted *P* *<* 0.05; Fig. 2a,b and Supplementary Table 7). While *Fusobacterium nucleatum* has been consistently associated with colorectal lesions across cohorts of diverse backgrounds^13,14^, in the LCPM cohort, *Fusobacterium nucleatum* absolute abundance was positively correlated with high fecal calprotectin levels (Spearman’s rank and Kendall’s tau correlations, adjusted *P* < 0.05; Fig. 2c, Extended Data Fig. 1 and Supplementary Table 8) and cancer progression (diagnosis groups) (KW test, *η*^2^ = 0.010, adjusted *P* = 1.84 × 10^−5^; phD test adjusted *P* = 8.80 × 10^−1^ for CTL versus ADE, adjusted *P* = 3.84 × 10^−7^ for CTL versus CRC and adjusted *P* = 3.84 × 10^−7^ for ADE versus CRC; Fig. 2c and Supplementary Table 7). However, after deconfounding for calprotectin only or combined BMI, moisture content and calprotectin, and neither absolute nor relative *Fusobacterium nucleatum* abundance were associated with diagnosis (generalized linear model analysis of variance (ANOVA), *n* = 547, *P* > 0.05; Extended Data Fig. 2).

### Multiple established CRC microbial markers are associated with transit time, intestinal inflammation and body mass index but not with CRC stages

The association of *Fusobacterium* abundance with fecal calprotectin urged us to investigate the influence of this confounder on previously reported CRC-associated genera, adding moisture content since it is the top microbiome covariate, and BMI, which showed differences among diagnosis groups.

To this end, we compiled a list of 89 CRC species-level markers from ten published cohorts^6,9,11,13,14,28–31^ (including 1,633 samples) and 67 genera-level markers from 15 cohorts^6–9,11–15,28–32^ (representing 4,439 samples). We used this compiled list of taxa as a criterion to test whether the CRC association of these taxa in our cohort is influenced by the target covariates. To reduce the impact of distinct statistical treatments, we downloaded the microbial profiles of nine out of ten studies at species level from the curated MetagenomicData^33^ resource and analyzed them using the statistical component of our pipeline.

**Fig. 3:**
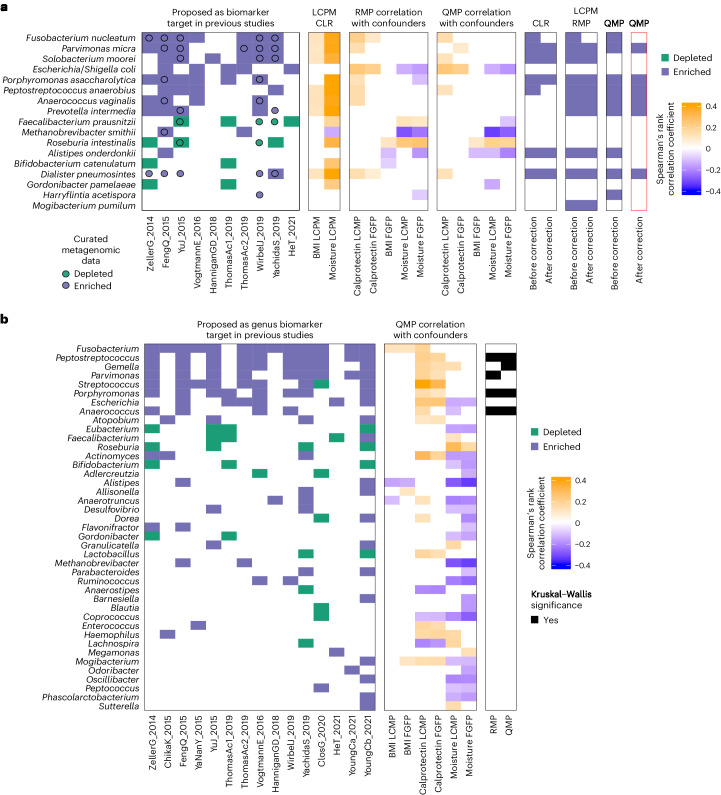
BMI, intestinal inflammation and moisture correlations with microbial biomarkers and CRC. **a**,**b**, Species (**a**) and genera (**b**) previously reported in association with CRC (blue and green represent enrichment or depletion; the squares indicate reported in corresponding publications, while circles represent our reanalysis of the MetaPhlAn 3.0 profiles generated from the curatedMetagenomicData^33^ of these cohorts using the statistical part of our pipeline). Graphic representation of Spearman’s rank correlation of pairwise analysis of fecal calprotectin, BMI, and moisture values against absolute species abundance (QMP) and RMP from the LCPM (*N* = 589) and FGFP (*N* = 1,045) cohorts (adjusted *P* < 0.05, Supplementary Table 8). The species enriched or depleted in relation to CRC diagnosis groups were tested using QMP, CLR and RMP data before (*n* = 589, two-sided KW test and Spearman’s rank correlation comparison, adjusted *P* < 0.05) and after controlling for microbiota covariates (before adjustment for BMI, calprotectin and moisture; generalized linear model ANOVA, adjusted *P* < 0.05). Source data

**Extended Data Fig. 3:**
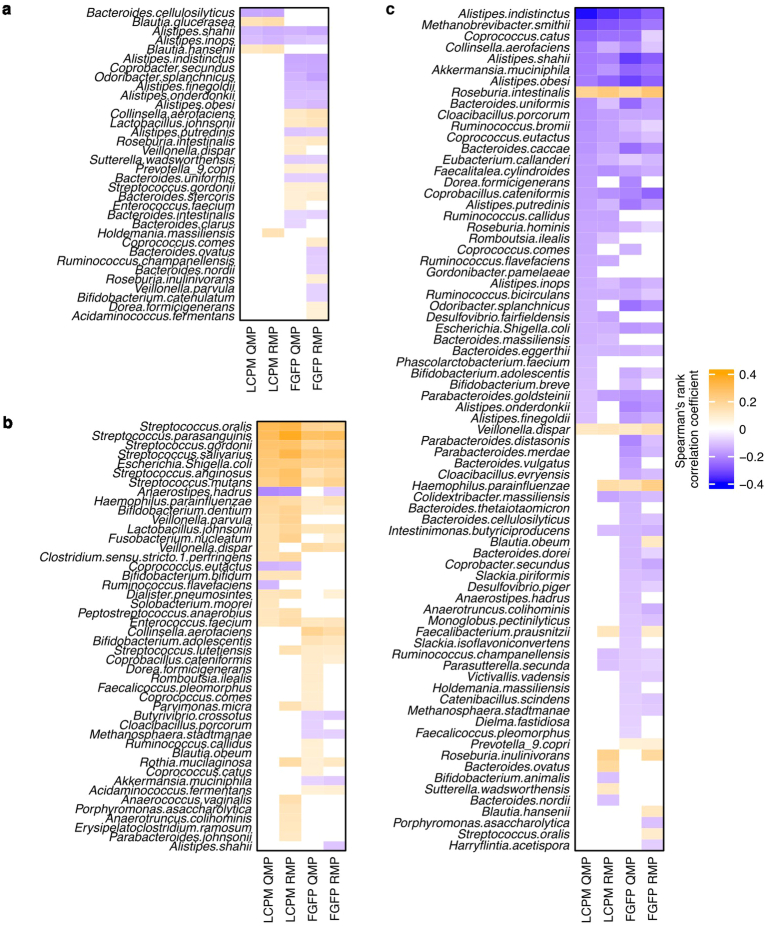
Spearman correlation between species abundance and microbiota covariates in the LCPM and FGFP cohorts. Two-sided Spearman’s rank correlation comparison between absolute species abundance (QMP) and relative abundance (RMP) from the LCPM (N = 589 samples) and FGFP (N = 1045 samples) cohorts and a, BMI b, faecal calprotectin and c, moisture content values. Spearman correlation adjP < 0.05 (QMP and RMP, Supplementary Table 8).

Spearman correlation between taxa abundances and the three focus covariates revealed strong associations between microbial targets and these confounders at the species (Extended Data Fig. 3a) and genus level (Fig. 3b). Most of these associations were replicated in an independent population cohort (FGFP), suggesting these associations are robust and not specifically linked to CRC (Extended Data Fig. 3). Moisture content, the known major covariate in microbiome studies^17^, is unsurprisingly associated with many taxa validated in both cohorts.

As we compiled the CRC-associated taxa from non-QMP studies, we conducted analyses using both RMP and QMP to assess whether confounder associations influence quantitative association of biomarkers or targets to diagnosis groups in LCPM. We found only 8% (6 out of 89) and 10% (9 out of 89) of species previously associated with CRC using QMP and RMP replicating after confounder control. *Anaerococcus vaginalis*, *Dialister pneumosintes*, *Parvimonas micra*, *Peptostreptococcus anaerobius*, *Prevotella intermeia* and *Porphyromonas asaccharolytica*, were identified by controlled QMP and RMP. Controlled QMP excluded *Fusobacterium nucleatum* and *Alistipes onderdonkii*, suggesting previous associations of these two species may be spurious (Fig. 3a).

We identified eight species previously linked to CRC (that is, using QMP and/or RMP), including *Fusobacterium nucleatum* *and Peptostreptococcus anaerobius*, to be associated with inflammation (Fig. 3 and Supplementary Tables 8 and 9). This association was previously reported for only three out of the eight taxa above (*Escherichia*, *Fusobacterium* and *Streptococcus*)^24^. Further validation of this association was conducted using the FGFP (Extended Data Fig. 3 and Supplementary Tables 8 and 9).

Recognizing that inflammation is a risk factor, not a requirement, for CRC progression, we further investigated markers associated with diagnosis groups in relation to inflammatory status. To this end, we focused on a subset of 340 samples, which, regardless of their CRC status, exhibited normal levels of calprotectin (fecal calprotectin under 50 μg g^−1^ (ref. ^34^)), indicating no evidence of local inflammation (112 CTL, 216 ADE and 12 CRC). Assessment of the 89 CRC species-level markers mentioned above confirmed that the association of three of the six replicating species (*Anaerococcus vaginalis*, *Prevotella intermedia* and *Porphyromonas asaccharolytica)* is independent of intestinal inflammation (Supplementary Table 10).

### Colonoscopy patients, with or without CRC, exhibit an excess of the Bacteroides2 enterotype

**Fig. 4:**
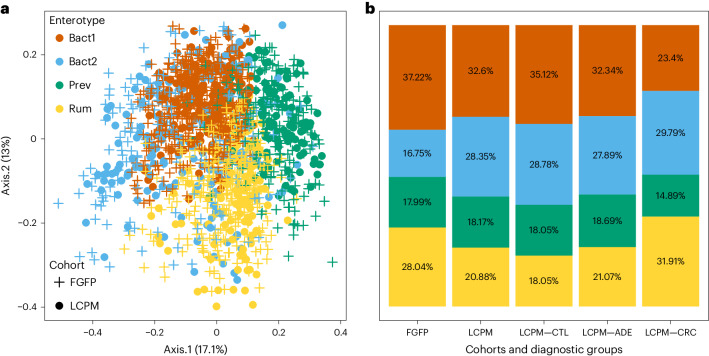
The Bact2 enterotype is enriched in patients referred for a colonoscopy (with and without colorectal lesions). **a**, PCoA of interindividual differences (BCD) in relative microbiota profiles of the LCPM cohort (*n* = 589 samples) using a cross-section of the Flemish population (*n* = 1,045 samples) as a background dataset. PCoA1 (Axis.1) and PCoA2 (Axis.2) respectively explained 13% and 17.1% of the variance of microbiota at the genus level. **b**, Enterotype distribution across the FGFP, LCPM and LCPM diagnosis groups (CTL, ADE and CRC), increased prevalence of the Bact2 enterotype in the three groups from the LCPM cohort (*n* = 589) as compared to FGFP samples (*n* = 1,045); pairwise two-sided test of equal or given proportions (*P* < 0.05). Source data

**Extended Data Fig. 4:**
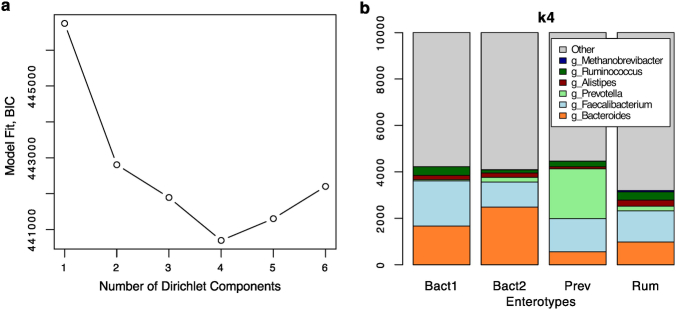
Enterotype stratification by DMM community typing. **a**, Identification of optimal number of clusters (Dirichlet components) in the LCPM cohort (n = 589) complemented with 1045 samples from the FGFP cohort, based on the Bayesian Information Criterion (BIC). **b**, Barplot representation of the average relative abundance of a few representative genera split into the four enterotypes identified by DMM community typing on the combined LCPM and FGFP cohorts (n = 1634).

To study the LCPM cohort in a population context, we enterotyped participants using Dirichlet multinomial mixtures (DMM) on a genus matrix against the background of microbial variation as observed in the FGFP samples (*n* = 1,045)^17^. Consistent with previous description of the Flemish population^23^, we identified four community types based on selecting the optimal number of clusters using the Bayesian Information Criterion (Fig. 4a,b and Extended Data Fig. 4), ‘Bacteroides1’ (Bact1), ‘Bacteroides2’ (Bact2), ‘Prevotella’ (Prev) and ‘Ruminococcaceae’ (Rum). The enterotype distribution was different between LCPM and FGFP (CS test, *χ*^2^ = 34.3, d.f. of 3, adjusted *P* = 1.7 × 10^−7^), but no differences were observed among diagnosis groups within the LCPM cohort (pairwise CS tests, adjusted *P* > 0.1). Pairwise comparisons of the prevalence of the dysbiotic Bact2 enterotype in the LCPM cohort diagnosis groups revealed that compared to the FGFP population, this enterotype was enriched in all CRC diagnosis groups (test of equal or given proportions, FGFP versus CTL: *χ*^2^ = 15.09, d.f. of 1, adjusted *P* = 1.1 × 10^−4^; FGFP versus ADE: *χ*^2^ = 18.93, d.f. of 1, adjusted *P* = 2.4 × 10^−5^; and FGFP versus CRC: *χ*^2^ = 4.34, d.f. of 1, adjusted *P* = 3.4 × 10^−2^). Although dysbiosis and CRC development were previously linked^13,35^, the high prevalence of this enterotype in the LCPM, even in samples from patients free of lesions, is unexpected. Consistent with previous reports^24,25^, the Bact2 enterotype in this group exhibited all hallmarks of dysbiosis: low cell count, low richness, higher calprotectin values, reduced butyrate producers and increased proinflammatory bacteria.

Additional categorical variables appeared associated with the Bact2 enterotype. They included antibiotic consumption (CS test, *χ*^2^ = 30.78, d.f. of 3, adjusted *P* = 2.1 × 10^−2^), current treatment with anti-inflammatory medications (CS test, *χ*^2^ = 30.78, d.f. of 3, adjusted *P* = 2.1 × 10^−2^), diabetes treatment (CS test, *χ*^2^ = 30.78, d.f. of 3, adjusted *P* = 3.3 × 10^−2^), recent diarrhea (last week) (CS test, *χ*^2^ = 30.78, d.f. of 3, adjusted *P* = 2.1 × 10^−2^), history of gallstones (CS test, *χ*^2^ = 30.78, d.f. of 3, adjusted *P* = 4.7 × 10^−2^) and recent use of laxatives (last week) (*χ*^2^ = 30.78, d.f. of 3, adjusted *P* = 4.2 × 10^−2^) (Supplementary Table 11).

## Discussion

While associations between the gut microbiota and CRC have been extensive, this is the first study using QMP and extensive metadata collection to systematically investigate microbiota covariates that potentially are masking or creating spurious associations between specific taxa and malignant transformation.

At first glance, this study yielded a gut microbial profile partially consistent with previous reports of CRC-associated taxa. Further analysis, however, suggested that many of the previously reported associations, including those of prominent biomarkers, such as *Fusobacterium (nucleatum),* are confounded by microbiota covariates. A total of 17 of 94 variables explained 6.7% of the observed variation. Of those, the moisture content had highest explanatory power (2.7%), greater than eight times that of the next covariate (IBD status). The explanatory power of fecal calprotectin was lower (0.2%) but significant; age and, most importantly, diagnosis groups were not.

Some associations were complex in nature. For example, BMI, consistent with previous reports, showed an association with both microbial composition^17,25^ and cancer progression^36^, while others, such as age, suggested to modify the BMI-association with cancer progression^37^, were not significant in this cohort.

Inflammation is a known risk factor for CRC^38^, but its effect size in shaping the cancer-associated microbiota is yet to be described. Fecal calprotectin is a well-documented marker of intestinal local inflammation^39,40^ and has been associated with cancer progression, probably having an effect on tumor development rather than on tumor initiation^41^. We observed participants with normal and elevated fecal calprotectin levels within each diagnosis group and covariate-controlled analysis of the LCPM cohort revealed that 8 and 19 CRC-associated markers, at the species and genus levels, respectively, associated with fecal calprotectin rather than with the diagnosis group. We replicated these observations in an independent cohort of apparently healthy individuals (FGFP).

High levels of fecal calprotectin have been associated with intestinal inflammatory pathologies^19^. However, when removing patients with IBD from our analysis, CRC diagnosis groups remained not significant, and the significance of *Fusobacterium nucleatum*, among other six species, was unaltered after differential abundance analysis. In patients with CRC, increased levels of fecal calprotectin (>50 µg g^−1^ stool^18,26^) are directly associated with tumor presence, as the level decreases after tumor resection^42^. Here, fecal calprotectin was increased in CRC, consistent with previous associations between malignant transformation, local inflammation^43^ and advanced tumor stages (T3 and T4)^42^. No difference in calprotectin levels was observed between CTL and ADE (mean 73.25 versus 70.24 µg g^−1^), suggesting that although no lesions are visible in the colon of the CTL group, they have a detectable level of local inflammation. The potential effect of local inflammation in shaping the colonic microbiota in the context of malignant transformation, or its potential confounding effect, remains largely obscure, as most studies surveying the association between gut microbiota and CRC, including meta-analysis^13,14^, do not control for local inflammation.

We argue that strict control of covariates is a must in any microbiota analysis assessing potential clinical associations, as for example, three of the species with repeated CRC association^11,13,14,28–30,32^, *Escherichia coli*, *Fusobacterium nucleatun* and *Parvimonas micra*, exhibit association with local inflammation, unfortunately uncontrolled for in previous studies, that may or may not be associated with cancer progression.

*Fusobacterium nucleatum* is one of the species that attracts more attention as there is a substantial body of work linking it to CRC^44^. In this study, *Fusobacterium* was enriched in patients with CRC. However, this apparent association disappears when the analysis is covariate controlled. Our study suggests that the association of *Fusobacterium nucleatum* to cancer may be driven by its association to intestinal inflammatory conditions; there are no differences in the abundance of *Fusobacterium nucleatum* across diagnostic groups once calprotectin is controlled for. These results suggest reassessment of the diagnostic utility of this marker. At the same time, our results do not mean that *Fusobacterium nucleatum* is not linked to CRC; they rather suggest that the reasons behind this association might be less straightforward than originally considered. They, thus, present a cautionary tale of the importance to control for covariates as the microbiome field moves forward. Given that inflammation is a risk factor for CRC but not a requirement^41^, potential use of *Fusobacterium nucleatum* as a marker of CRC development could fail to identify those cases of inflammation-independent cancer progression. While not yet commercialized, there are already publications proposing the use of microbial markers, including *Fusobacterium nucleatum*, for CRC screening^7,45^, which, in light of our results, raises concerns as uncontrolled variables may be obscuring actual biological mechanisms. We present evidence that purported CRC biomarkers, even those replicated in multiple studies, may suffer from the compounding or confounding effect of covariates, which in addition to the use of nonquantitative signals, may result in misleading conclusions on what diagnostic signals really mean—complicating the path towards potential clinical applications.

BMI, in combination or independent of inflammation, has been independently associated with changes in the gut microbiota^46^, which in turn are associated with increased risk of CRC^47^. Yet, microbial dysbiosis by itself does not explain the higher risk of colon cancer observed in the obese population^48^, indicating that the underlying process that associates obesity and CRC is more complex and demands further investigation.

Among four described gut enterotypes, the Bact2 enterotype is defined as a dysbiotic microbial profile^24,25^. Bact2 enrichment is observed in obesity^25^ and in conditions such as PSC (Primary sclerosing cholangitis) and IBD^24^, further supporting the potential disease association of this enterotype. The analysis of the LCPM cohort revealed an excess of the Bact2 enterotype across all diagnosis subgroups, regardless of BMI.

Increased Bact2 prevalence in the no-lesions group compared to FGFP is particularly striking. While patients in the CTL group have no observable lesions, they may be considered at increased risk for colorectal perturbations based on clinical referrals (blood loss in the stool, familiar risk to colonic lesion and so on) that warranted colonoscopies—something that might also be reflected by their Bact2 enterotype. Of importance, ‘healthy’ biopsies included in CRC microbiome studies are often selected using colonoscopies with a negative result as the main criterium, posing a potential problem, as no other markers of colonic health are considered to qualify these healthy individuals. The reasons for the appearance of Bact2 in the no-lesion group are multifold, but these findings suggest that such individuals, while representing a useful category for biomarker discovery, may harbor an unhealthy gut ecosystem, from a microbial point of view.

There is a plethora of variables identified as modifiers of the gut microbiota. Yet, covariate control is far from standard and notably absent from most association studies. As intestinal microbial taxa are being nominated as potential biomarkers of malignant transformation, it is imperative to explore the influence of microbiota covariates as potential confounders or compounders of observed associations. Rather than denying previous associations, our analysis emphasizes the need for covariate-controlled analysis for any microbiota study aiming to establish clinical associations, as these covariates by themselves may explain most of the stool microbiota variation, independent of CRC status.

Out of the multiple taxa previously associated with CRC, six species remain significant after strict control of covariates in this quantitative cohort. Without denying other potential biomarkers, further studies are warranted on *Anaerococcus vaginalis*, *Dialister pneumosintes*, *Parvimonas micra*, *Peptostreptococcus anaerobius*, *Prevotella intermedia* and *Porphyromonas asaccharolytica*, as their reported association to CRC^6,7^ is robust enough to remain independent of the method. Our data present a strong argument in favor of revisiting potential microbial associations with clinical phenotypes to ensure that the purported associations are not driven by uncontrolled covariates warranting further follow up of the mechanisms underlying these associations. Refining the approaches to discover microbial biomarkers will undoubtedly impact the microbiota field, facilitating the path towards the much-coveted clinical applications.

## Limitations

We aim to identify taxa associated with malignant colonic transformation. While our cohort includes a set of participants without lesions, we make no claim that these are healthy controls, as there is an apparent increased incidence of gut dysbiosis in this group. Considering that all participants in this study had a medical need for a colonoscopy, there is an implicit increased risk to CRC. Thus, the present study cannot rule out that the group without polyps is undergoing potential molecular or cellular changes that are not detectable via colonoscopy. In addition, as this is a cross-sectional study, the term cancer progression is an extrapolation of what is seen at cancer development stages (operationalized here as diagnosis groups). We cannot rule out potential particularities of our cohort that may be contributing to our observations, as most studies do not report sufficient metadata for us to compare across cohorts. It is important to consider that certain taxonomic groups may not even be represented in current databases, and specific microbial species may require longer hypervariable regions or alternative sequencing approaches to achieve accurate species-level identification. Nonetheless, the V4 region for our cohort seems to be able to resolve species taxonomy of the biomarkers previously associated with CRC, as we show for the case of *Fusobacterium*.

Furthermore, it has been proposed that the potential diagnostic value of colonic microbial profiles goes beyond bacteria, as fungal and viral species have been proposed as CRC biomarkers^49^. We recognize that multidomain approaches to discover CRC biomarkers and longitudinal prospective studies to better study the dynamics of cancer progression are warranted to comprehensively inform cancer detection and treatment.

## Methods

### Participant recruitment

The LCPM project was an observational cross-sectional survey for which procedures were approved by the medical ethics committee of the UZL (ethical approval number S57084). Between 2017 and 2018, we recruited patients through the study nurse following a standardized procedure. Briefly, we invited patients scheduled for lower gastrointestinal endoscopy or abdominal surgery for CRC removal at the UZL were invited. After explaining the research project and if they expressed their agreement, participants signed an informed consent, and no compensation was offered. A set of stool sample collection material was provided.

Each patient completed an extensive questionnaire containing information about the date of sample collection, the consistency of the stool, diet, antibiotics usage, clinical symptoms or disease among other variables^17^, as well as an extensive medical and clinical questionnaire using the Websurvey service of KU Leuven.

As a validation cohort we included the FGFP^17^, a population-wide microbiota monitoring effort, representing one of the largest and best characterized fecal microbiota database currently available. Its extensive metadata including health and lifestyle allowed the identification of 69 factors associated with microbiota variation (microbiota covariates). The QMP transformation was conducted in parallel, with the same protocol, for both the FGFP and the LCPM cohorts.

### CRC status classification

We invited patients referred for colonoscopy or colectomy to participate in the study. Those that consented were instructed to collect a stool sample at home, which was kept frozen using a sample kit provided by the research team. Upon completion of the medically necessary procedures (colonoscopy or colon resection), we stratified study participants into three diagnosis groups according to their clinical phenotype: (1) patients without evidence of lesions, (2) patients with polyps (*n* < 10 and size between 6 and 10 mm) (ADE) and (3) patients with CRC. Patients whose clinical presentation did not fit any of these three groups were excluded from the study. Once the participants were included in the corresponding groups, extensive metadata was collected from their medical records as stated in the informed consent.

### Sample collection

The stool samples of patients from UZL were collected as part of the LCPM project using aliquot ready mat without any buffer or preservative (Supplementary Fig. 1). The samples were kept at −20 °C freezers at the patients’ homes and brought to our laboratory on icepacks. Upon arrival, samples were stored in the Raes’ Lab at −80 °C until further analysis. Each stool sample had a temperature logger to make sure that, during the storage at home or transport to the laboratory, low stable temperature was maintained.

### Stool sample analyses

#### Microbial load measurement by flow cytometry

We determined microbial loads of stool samples of LCPM patients following published procedures^23^. We performed cell counting for all other samples in triplicate. Briefly, we dissolved 0.2 g frozen (−80 °C) aliquots in physiological solution to a total volume of 100 ml (8.5 g l^−1^NaCl; VWR International). Subsequently, the slurry was diluted 1,000 times. The samples were filtered using a sterile syringe filter (pore size of 5 μm; Sartorius Stedim Biotech). Next, we stained 1 ml of the microbial cell suspension obtained with 1 μl SYBR Green I (1:100 dilution in dimethylsulfoxide; shaded for 15 min of incubation at 37 °C; 10,000 concentrate, Thermo Fisher Scientific) and monitored fluorescence events using the FL1 533/530 nm and FL3 >670 nm optical detectors of the C6 Accuri flow cytometer (BD Biosciences). In addition, forward and sideward scattered light was collected. The BD Accuri CFlow (v.1.0.264.21) software was used to gate and separate the microbial fluorescence events on the FL1/FL3 density plot from background events Supplementary Fig. 2. A threshold value of 2,000 was applied on the FL1 channel. We evaluated the gated fluorescence events on the forward and sideward density plot, as to exclude remaining background events. We kept instrument and gating settings identical for all samples as described previously^24^. Based on the exact weight of the aliquots analyzed, we converted cell counts to microbial loads per gram of fecal material.

#### Fecal moisture content

We determined moisture content as the percentage of mass loss after lyophilization from 0.2 g frozen aliquots of nonhomogenized fecal material (−80 °C) as described previously^24^.

#### Fecal calprotectin measurement

We quantified fecal calprotectin concentrations using the fCAL ELISA Kit (Buhlmann). For patients and FGFP participants, we conducted analyses on frozen fecal material (−80 °C) as described previously^24^.

### Microbiota phylogenetic profiling

#### DNA extraction and sequencing data preprocessing

The fecal microbiota profile of the FGFP cohort was described previously^17^. For fecal DNA extraction and microbiota profiling of the new cohort, we followed the same protocols^17^.

**Extended Data Fig. 5:**
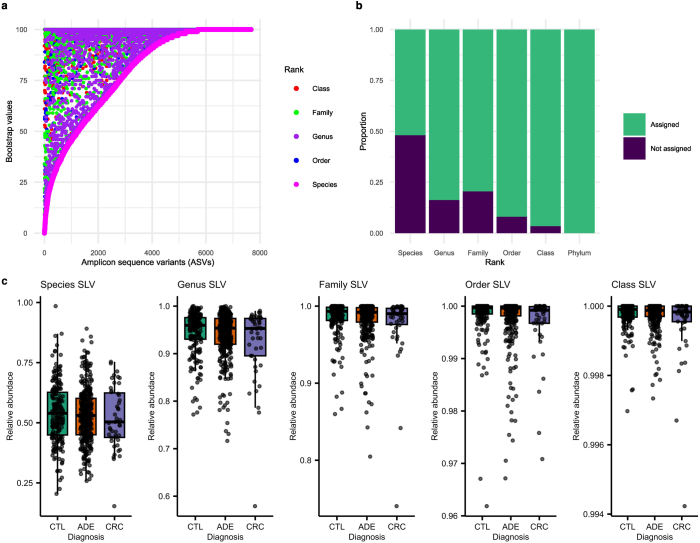
Taxa assignation performance of the V4 amplicon marker in the LCPM. **a**, Bootstrap values distribution across different ranks, **b**, Proportion of ASVs assigned from species to phylum, **c**, Proportion of ASVs assigned from species to phylum to each sample. The whiskers extend from the quartiles to the last data point within 1.5× of the interquartile range, with outliers beyond. The figure below (Panel a) illustrates our taxa assignation performance, showing that more than half of the ASVs were assigned to species level with bootstrap values above 80. Panel b shows the ASV assignation proportions from phylum (100%) to species level (50%). A comparison of proportions of ASVs assigned from each sample at different taxonomic levels revealed no significant differences in the distributions of assigned ASVs per sample across diagnosis groups, as indicated in panel c (KW test, p-values > 0.05). The center of the box plot represents the median value of the data, and the whiskers extend from the quartiles to the last data point within 1.5× of the interquartile range, with outliers beyond.

The bacterial profiling was carried out as described previously^50^. Briefly, we extracted nucleic acids from frozen fecal aliquots using the MagAttract PowerMicrobiome DNA/RNA kit (Qiagen). We modified the manufacturer’s protocol by the addition of a heating step at 90 °C for 10 min after vortexing and excluding the steps where DNA is removed. For bacterial and archaeal characterization, we used 16S ribosomal RNA primers 515F (5′-GTGYCAGCMGCCGCGGTAA-3′) and 806R (5′-GGACTACNVGGGTWTCTAAT-3′) targeting the V4 region. These primers were modified to contain a barcode sequence between each primer and the Illumina adapter sequences to produce dual-barcoded libraries from the extracted DNA (dilution 1:10) in triplicate. Deep sequencing was performed on a MiSeq platform (2 × 250 paired end (PE) reads, Illumina). We randomized all samples and negative controls (polymerase chain reaction (PCR) and extraction controls) taken along for sequencing. After demultiplexing with sdm as part of the LotuS pipeline (v. 1.60)^51^ without allowing for mismatches, we further analyzed fastq sequences per sample using DADA2 pipeline (v. 1.6)^52^. Briefly, we removed the primer sequences and the first ten nucleotides after the primer. After merging paired sequences and removing chimeras, we assigned taxonomy using formatted Silva set ‘SLV_nr99_v138.1’. We performed taxonomic assignments at the domain, class, order, family, genus and species levels were performed using the ‘assignTaxonomy’ function from the DADA2 R library, by a naive Bayesian classifier method with a minimum bootstrap confidence of 50, using the ‘silva_nr99_v138.1_wSpecies_train_set.fa.gz’ training database (Extended Data Fig. 5). Deep sequencing was performed on a MiSeq platform from the DADA2 R library with the formatted Silva SSU database ‘silva_species_assignment_v138.1.fa.gz’ to obtain species assignments for the amplicon sequence variants (ASVs). We labeled any unassigned ASVs at any taxonomic level, with the prefix ‘uc’ along with the assigned taxonomic level (not species level) to avoid the lack of labels.

Before the analyses, we removed sequences annotated to the class Chloroplast, family mitochondria or unknown archaea and bacteria from eukaryotic origin. phyloseq (v. 1.36.0)^53^ and MicroViz (v. 0.11.0)^54^ libraries were used for data curation and figure generation.

#### RMP

For the relative microbiome matrix, we transformed ASV counts to relative abundances. In other words, we divided ASV counts by the total counts of ASV per sample. We agglomerated ASV to species level using the phyloseq (v. 1.36.0)^53^ function ‘tax_glom’.

#### RMP (CLR)

We agglomerated ASV to the species level, and the abundance matrix was centered log-ratio (CLR)-transformed using ‘codaSeq.clr*’* in the CoDaSeq (v. 0.99.6)^55^ using the minimum proportional abundance detected for each taxon for the imputation of zeros.

#### Workflow Assessment

**Extended Data Fig. 6:**
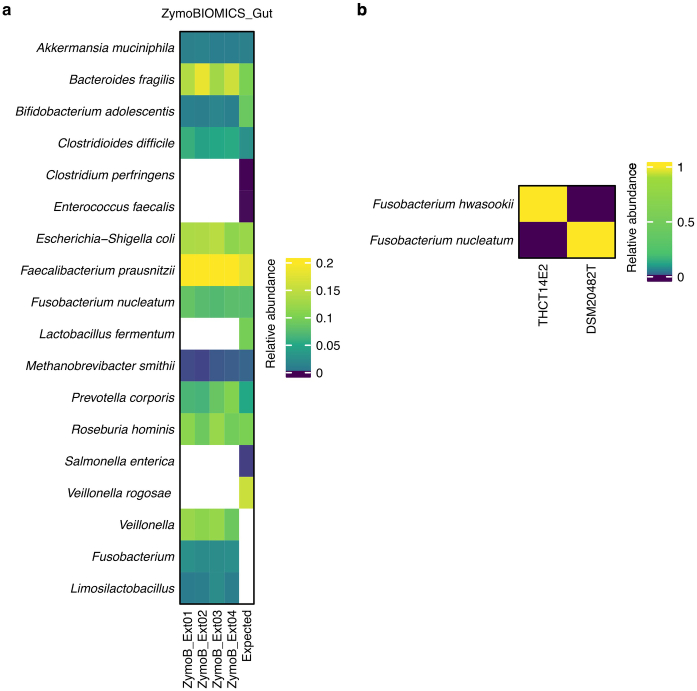
Performance of our methodology in small communities and isolated microorganisms. **a**, Species composition of the ZymoBIOMICS gut controls, ten successfully identified species and **b**, two Fusobacterium species: Fusobacterium hwasookii (THCT14E2) and Fusobacterium nucleatum (DSM 20482T) were successfully identified using our methodology.

We conducted a workflow assessment using (1) a commercial mock community, ZymoBIOMICS Gut, and (2) two *Fusobacterium* species: *Fusobacterium hwasookii* (THCT14E2) and *Fusobacterium nucleatum* (DSM 20482T). The assessment followed our standard methods, involving the amplification, sequencing and analysis of the extracted DNA. This evaluation aimed to assess the performance of our full methodology, as depicted in Extended Data Fig. 6.

#### Quality control assessment for amplicon sequencing data (16S rRNA) using RMP

**Extended Data Fig. 7:**
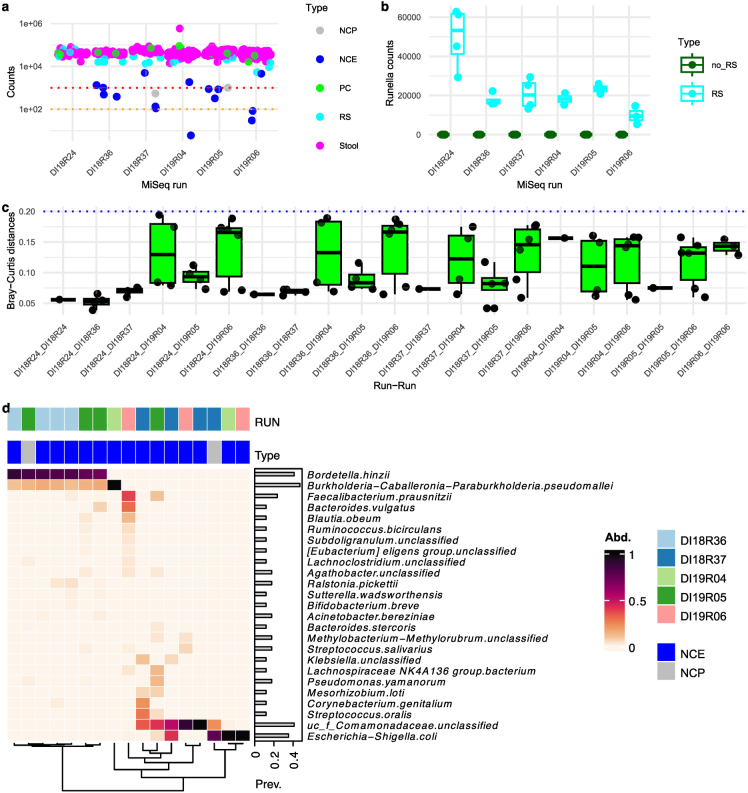
Quality control assessment for amplicon sequencing data (V4 16S rRNA gene). **a**, The obtained reads for each sample are shown after processing with DADA2 (red and orange dashed lines represent 10, 000 and 1,000 reads, respectively; NCP: PCR negative control, NCE: DNA extraction Negative control, PC: positive control, and RS: Runella slithyformis control). **b**, Sequencing controls reveal the absence of barcode crosstalk. RS sequences serve as a marker for barcode crosstalk during sequencing. The absence of RS sequences in the samples without RS (no_RS) ruled out barcode crosstalk during the sequencing or PCR setup procedures. **c**, BCD among technical replicates demonstrating reproducibility. Pairwise comparisons between PC samples within and among MiSeq runs showed values under 0.2 (depicted by the pointed blue line). The center of the box plot represents the median value of the data, and the whiskers extend from the quartiles to the last data point within 1.5× of the interquartile range, with outliers beyond. **d**, Species composition of negative controls is presented, indicating the relative abundance and prevalence of the top 20 species. None of the species detected with differential abundance using QMP, RMP or CLR were found as background contaminants. Non-significant differences in bacteria composition were observed among DNA sequencing runs (Padj > 0.05, pairwiseAdonis test). A full list of detected species is available in Supplementary Table 12. Of note, DI18R24 is not shown as the negative controls (NCE and NCP) did not produce reads.

In short, we sequenced all samples in six MiSeq runs (Extended Data Fig. 7a). Per each run, we used a set of internal controls to identify: 1) Technical variation within and between runs 1) Contamination events during the DNA extraction, 2) Contamination events during the amplification and sequencing procedures and, 3) Carry-over contamination at the sequencing facility and barcode crosstalk.

We amplified all samples, including biological material (stool samples), positive controls (DNA from a stool sample previously profiled and RS: nonhuman gut bacteria strain ‘*Runella slithyformis’*), negative controls (negative control of extraction (NCE) and negative control during PCR (NCP)) in triplicate using a unique barcode combination, while omitting several barcode combinations to control for primer synthesis cross contamination. We used *Runella slithyformis* in duplicate within each sequencing library to detect barcode crosstalk during the sequencing procedure (Extended Data Fig. 7b). This genus is not detected in human gut samples; therefore, we expected no *Runella slithyformis* reads in any of the stool samples analyzed. We determined technical variation based on the BCD of positive control samples (Extended Data Fig. 7c). Finally, we included NCEs along the whole process from extraction to bioinformatic analysis. For amplification and sequencing contamination^56^, we used NCP and NCE (Extended Data Fig. 7d and Supplementary Table 12), and for carry-over contamination events, we used a different set of barcode combinations in consecutive MiSeq runs^56^.

#### QMP

We built the QMP matrix as described previously^23^. In brief, we downsized samples to even sampling depth, defined as the ratio between sampling size (16S rRNA gene copy number-corrected sequencing depth) and microbial load (the average total cell counts per gram of frozen fecal material; Supplementary Table 2). We imputed 16S rRNA genome copies (GC) numbers using RasperGade16S (v. 0.0.1)^57^, a new tool that utilizes a heterogeneous pulsed evolution model for predicting 16S rRNA GC. It not only predicts the GC but also provides confidence estimates for the predictions^57^. We used a minimum rarefied read count of less than 150 for QMP analyses. We converted rarefied ASV abundances into numbers of cells per gram. The QMP matrices had a final size of 589 samples for the study cohort and 1,045 samples for the FGFP validation cohort^17^. We agglomerate the QMP matrix at ASV level to species level using the phyloseq (v. 1.36.0)^53^ function ‘tax_glom’. We used the resulting species QMP matrix for the main analysis.

### Statistical analysis

We performed all statistical analyses with R (Version 4.2.1, RStudio v.2022.12.0 + 353, 86_64-apple-darwin17.0 (64-bit)) and packages phyloseq (v. 1.36.0)^53^, vegan (v. 2.6.2)^58^, coin(v. 1.4.2)^59^, effectsize (v. 0.8.3), vcd(1.4.11)^60^*,* DirichletMultinomial(v. 1.34.0)^61^, pairwiseAdonis (v. 0.4.1) and microbiome (v. 1.14.0)^62^. We used nonparametric statistical tests for robust comparisons among unbalanced groups. For multiple testing, we corrected all *P* values using the Benjamini–Hochberg method (reported as adjusted *P*) as appropriate on lists (*n* > 1) of features (for example, taxa–metadata or metadata–metadata associations) and also when performing multiple pairwise group (*n* > 2) comparisons (for example, KW test with phD test).

#### Fecal microbiota derived features and visualization

We visualized microbiota interindividual variation by PCoA using BCD on the species QMP matrix^24,25^. All the rest of the microbiota derived features were calculated based on QMP. We determined the contribution of metadata variables to microbiota community variation (effect size) of each of 94 variables by dbRDA on a species-level BCD with the capscale function in the vegan package^58^. We visualized absolute abundance species as log10 (abundance +1). This was the same for relative abundance.

#### Microbiota and physiological features associations

We excluded from analyzes any taxa unclassified at the species level or present in less than 5% of samples per each diagnosis group (Supplementary Table 6). We used Spearman correlations for rank–order correlations, between continuous variables complemented by Kendall’s tau correlation, including species abundances, calprotectin values and moisture content. We used the Mann–Whitney *U*-test to test median differences of continuous variables between two different groups. For more than two groups, for example, for differential abundance analysis for QMP and RMP taxa versus diagnosis groups, we used the KW test with phD test. For differential abundance analysis among diagnosis groups and bacteria species abundances from CLR transformed data, we performed an ANOVA test.

We evaluated statistical differences in the proportions of categorical variables (enterotypes) between patient groups using pairwise CS tests. We tested for deconfounded microbiota contributions to the diagnosis groups variable by using a nested model comparison (ANOVA) of generalized linear models as follows:

$$\begin{array}{l}[{\rm{null}}\,{\rm{model}}]\,{\rm{glm}}0={\rm{rank}}({\rm{abundance}})+{\rm{rank}}({\rm{calprotectin}})\\\qquad\qquad\qquad\quad\quad+{\rm{rank}}({\rm{moisture}})+{\rm{rank}}({\rm{BMI}})\end{array}$$

[alternative model] glm1 = rank(abundance) + rank(calprotectin) + rank(moisture) + rank(BMI) + diagnosis, where the diagnosis groups were recoded as 1, 2 and 3 for patients without evidence of CTLs, patients with polyps and patients with CRC, respectively. We treated this variable as a continuous variable, translating the directional increase in disease progression, from healthy to lesions, in the colonic mucosa. For the nested model comparison, we used taxa abundances (quantitative or relative) as explanatory variables, the diagnosis groups variable as response variable and BMI, fecal calprotectin and moisture as covariates. Additionally, we employed rank-transformed modeling to perform nonparametric testing on data that is not normally distributed, such as species abundances.

#### Previous reported CRC microbial markers

To compile a list of published CRC markers that would define taxa that should be tested against covariates in our data set, we conducted a PubMed search query using the keywords ‘CRC AND microbiome AND stool AND human AND biomarkers’. We found ten studies that met our inclusion criteria, namely: (1) a sample size minimum of 60 and (2) the CRC biomarker described at the species level, with statistical significance, in the main text of the publication. We included this list of published biomarkers in our correlation analysis between taxa and the three main covariates (fecal calprotectin, BMI and moisture) within the LCPM cohort. A similar procedure was followed at the genus level, which included 15 studies found in our PubMed search.

#### CRC microbial markers identification

**Extended Data Fig. 8:**
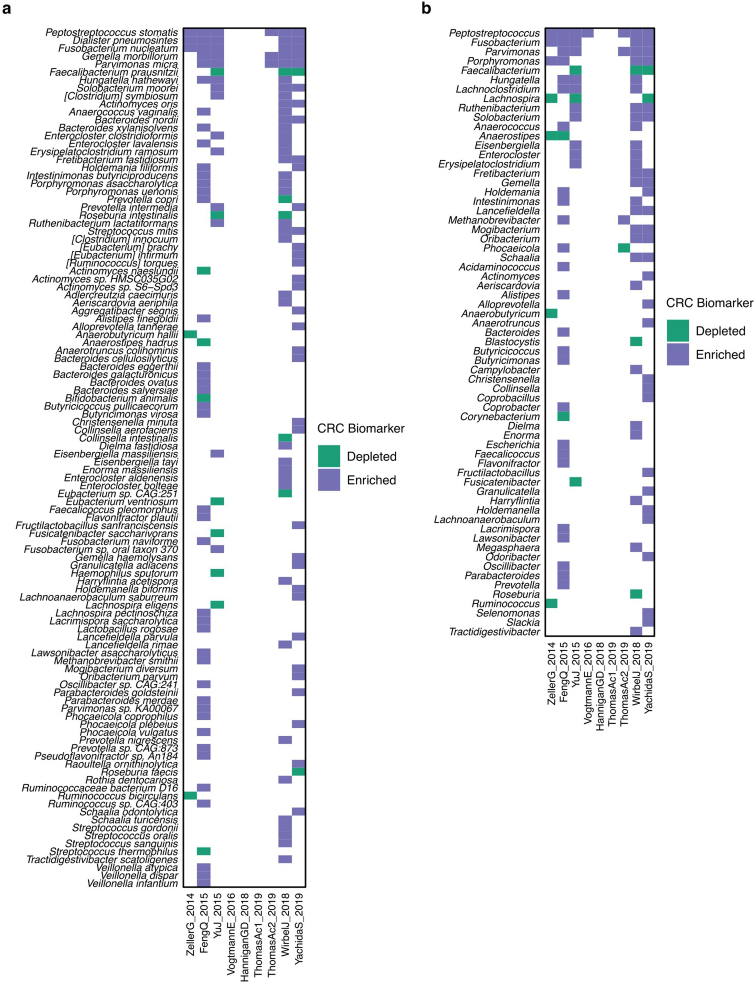
Species and genera associated with CRC on a subset of the curatedMetagenomicData. After performing our differential abundance procedure on the MataPhalAn 3.0 profiles downloaded from the curatedMetagenomicData, 108 species (**a**) and 63 genera (**b**) were identified across the 9 metagenomics datasets.

We performed differential abundance analyzes on nine different CRC shotgun datasets as part of ‘curatedMetagenomicData’^33^ using MetaPhlAn 3.0 profiles to compare the results while controlling for potential differences arising from the classification tools and statistical methods used in each independent study. The results of the meta-analysis are presented in Extended Data Fig. 8 and Supplementary Table 13.

#### Enterotyping and visualization

Using the genus matrix (agglomerated and downsized to 10,000 reads), we enterotyped and calculated observed genus richness^53^, as already reported for previous studies^24,25^. For enterotyping (or community typing) based on the DMM approach we used R as described previously^61^. We performed enterotyping on a combined genus-level abundance RMP matrix including LCPM samples compiled with 1,045 samples originating from the FGFP^17^. The optimal number of Dirichlet components based on the Bayesian information criterion was four. The four clusters were named ‘Bact1’, ‘Bact2’, ‘Prev’ and ‘Rum’, as described previously^23^.

### Reporting summary

Further information on research design is available in the Nature Portfolio Reporting Summary linked to this article.

## Online content

Any methods, additional references, Nature Portfolio reporting summaries, source data, extended data, supplementary information, acknowledgements, peer review information; details of author contributions and competing interests; and statements of data and code availability are available at 10.1038/s41591-024-02963-2.

## Supplementary information

Supplementary Information Supplementary Figs. 1 and 2 and Tables 1–14.

Reporting Summary

Supplementary Tables 1–14 Supplementary Table 1. Reasons for the colonoscopy referral of the LCPM cohort. Supplementary Table 2. LCMP cohort variable names, 95 variables plus enterotypes. Supplementary Table 3. Associations between continuous variables and cancer progression (KW test with phD tests. *N* is specified for each test, and statistical significance was derived from two-sided testing and adjusted for multiple testing (adjusted *P*, Benjamini–Hochberg method)). Supplementary Table 4. Associations between categorical variables and cancer progression (two-sided CS test; statistical significance was derived from two-sided testing and adjusted for multiple testing (adjusted *P*, Benjamini–Hochberg method)). Supplementary Table 5. Microbiome variation in the LCMP cohort. Independent and cumulative contribution of metadata variables to species-level microbiome variation (dbRDA and stepwise dbRDA; false discovery rate by Benjamini–Hochberg). Cumulative explanatory power and significance level of the included variables are reported. Supplementary Table 6. List of species excluded and included from the analysis. Supplementary Table 7. Differences in absolute (QMP) and relative (RMP) species abundances over diagnostic groups LCMP cohort (*n* = 589, KW, phD test; statistical significance was derived from two-sided testing and adjusted for multiple testing (adjusted *P*, Benjamini–Hochberg method)). Supplementary Table 8. Associations between species abundances (QMP and RMP) and BMI, intestinal calprotectin and moisture in the LCPM cohort (*n* = 589, Spearman and Kendall’s tau; statistical significance was derived from two-sided testing and adjusted for multiple testing (adjusted *P*, Benjamini–Hochberg method)). Supplementary Table 9. Associations between species abundances (QMP and RMP) and BMI, intestinal calprotectin and moisture in the FGFP cohort (*n* = 1,045, Spearman; statistical significance was derived from two-sided testing and adjusted for multiple testing (adjusted *P*, Benjamini–Hochberg method)). Supplementary Table 10. Differences in absolute (QMP) and relative (RMP) species abundances over diagnostic groups in the LCMP cohort subset with normal levels of fecal calprotectin (*n* = 340 (112 PWoL, 216 PWP and 12 PWT, KW and adjusted for multiple testing (adjusted *P*, Benjamini–Hochberg method)). Supplementary Table 11. Associations between categorical variables and enterotype distribution (two-sided CS test; statistical significance was derived from two-sided testing and adjusted for multiple testing (adjusted *P*, Benjamini–Hochberg method)). Supplementary Table 12. Full list of the species detected in the negative controls (NCE and NCP). Supplementary Table 13. Differences in relative abundances of species profiles from MetaPhlAn 3.0 between CRC and controls from nine published CRC cohorts from the curatedMetagenomicData (*n* = 1,254, two-sided Wilcoxon signed-rank test and adjusted for multiple testing (adjusted *P*, Benjamini–Hochberg method)). Supplementary Table 14. Absolute taxonomic abundances at species level in the LCMP cohort (*n* = 589).

## Source data

Source Data Fig. 1 Statistical source data.

Source Data Fig. 2 Statistical source data.

Source Data Fig. 3 Statistical source data.

Source Data Fig. 4 Statistical source data.

## Data Availability

Raw amplicon sequencing data and metadata reported in this study have been deposited in European Nucleotide Archive with accession code EGAS00001007413. FGFP 16S rRNA gene sequencing data and metadata are available at the European Genome-phenome Archive (EGAS00001003296). The diagnosis metadata and processed microbiome data required for the reanalysis are provided as Supplementary Tables 1 and 14, respectively. Formatted Silva set ‘SLV_nr99_v138.1’ files were downloaded from Zenodo via https://zenodo.org/records/4587955/files/silva_nr99_v138.1_wSpecies_train_set.fa.gz?download=1 (silva_nr99_v138.1_wSpecies_train_set.fa.gz)^63^ and https://zenodo.org/records/4587955/files/silva_species_assignment_v138.1.fa.gz?download=1 (silva_species_assignment_v138.1.fa.gz)^63^. The nine CRC cohort MetaPhlAn 3.0 profiles were collected from curatedMetagenomicData, study names: FengQ_2015, HanniganGD_2017, ThomasAM_2018a, ThomasAM_2018b, VogtmannE_2016, WirbelJ_2018, YachidaS_2019 and YuJ_2015, ZellerG_2014 (10.18129/B9.bioc.curatedMetagenomicData). Source data are provided with this paper.

## Code availability

Analysis codes are available via Github at https://github.com/raeslab/QMP-Microbiome-CRC-confounders.
