# Supplementary material for: Microbiome confounders and quantitative profiling challenge predicted microbial targets in colorectal cancer development
Source: Nat Med. 2024 Apr 30;30(5):1339–48. doi: 10.1038/s41591-024-02963-2 (PMC11108775; doi:10.1038/s41591-024-02963-2)
Supplement: Supplementary file 1 — Supplementary Figs. 1 and 2 and Tables 1–14. [file 41591_2024_2963_MOESM1_ESM.pdf]

nature medicine

Article

https://doi.org/10.1038/s41591-024-02963-2

Microbiome confounders and quantitative profiling challenge predicted microbial targets in colorectal cancer development

In the format provided by the authors and unedited

Supplementary Information

Microbiome confounders and quantitative profiling challenge predicted microbial targets in colorectal cancer development

Raúl Y. Tito[1,2,*], Sara Verbandt[3,*], Marta Aguirre Vazquez[3], Leo Lahti[1,4], Chloe Verspecht[1,2], Verónica Lloréns-Rico[1,2,5], Sara Vieira-Silva[1,6,7], Janine Arts[8], Gwen Falony[1,2,6], Evelien Dekker[9], Joke Reumers[10], Sabine Tejpar[3,**], Jeroen Raes[1,2,**,§]

Correspondence and requests for materials should be addressed to Jeroen Raes.
Email: jeroen.raes@kuleuven.be

This file includes:
Supplementary Figure 1 and 2
Other supplementary material for this manuscript:
Supplementary Tables 1 to 14.

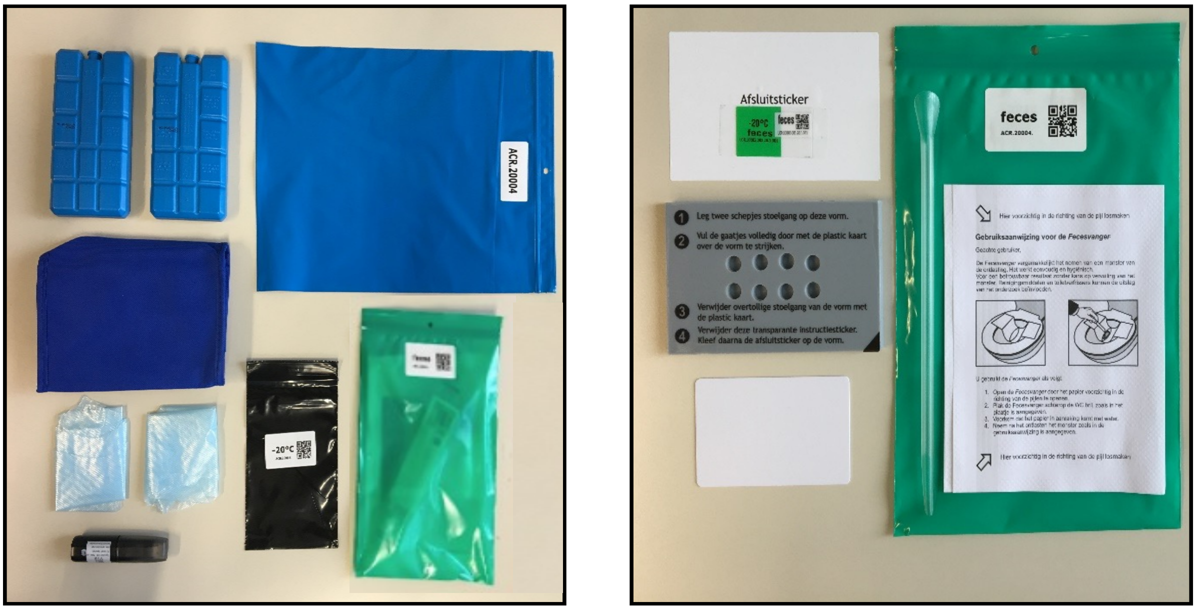

Supplementary Fig. 1 Stool sampling kit. Sample collection material provided to each of the participants of the LCPM cohort. Plastic gloves, bags, plastic scooper, and aliquot ready mat for the stool sample collection.

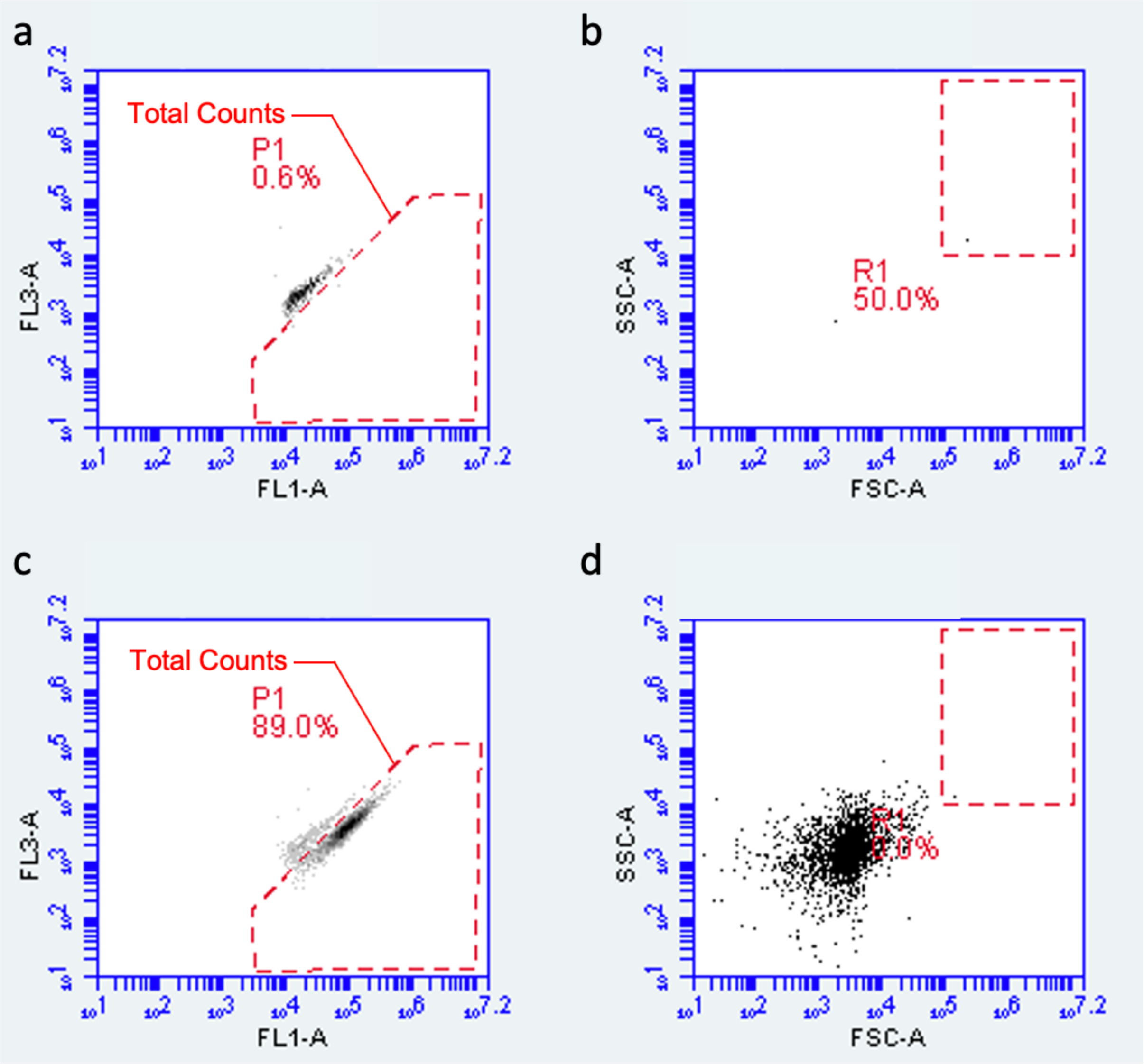

Supplementary Fig. 2 Illustration of flow cytometry gating strategy. A fixed gating and staining/approach was applied[25]. Both blank and sample solutions were stained with SYBR Green I. **a**, The FL1-A/FL3-A acquisition plot of a blank sample (0.85% w/v physiological solution) with gate boundaries indicated. A threshold value of 2,000 was applied on the FL1 channel. **b**, Secondary gating was performed on the FSC-A/SSC-A channels to further discriminate between debris or background and microbial events. c, d, FL1-A/FL3-A count acquisition of a faecal sample (**c**) with secondary gating on FSC-A/SSC-A channels based on blank analyses (**d**). Total counts were defined as events registered in the FL1-A/FL3-A gating area, excluding debris or background events observed in the FSC-A/SSC-A R1 gate. The flow rate was set at 14 microliters per minute and the acquisition rate did not exceed 10,000 events per second. Each panel reflects events registered over the course of a 30-s acquisition period. Cell counts were determined in duplicate starting from a single biological sample.
