# Supplementary material for: Microbiome confounders and quantitative profiling challenge predicted microbial targets in colorectal cancer development
Source: Nat Med. 2024 Apr 30;30(5):1339–48. doi: 10.1038/s41591-024-02963-2 (PMC11108775; doi:10.1038/s41591-024-02963-2)
Supplement: Supplementary file 2 — Reporting Summary [file 41591_2024_2963_MOESM2_ESM.pdf]

nature portfolio

Corresponding author(s): Jeroen Raes

Last updated by author(s): Mar 21, 2024

Reporting Summary

Nature Portfolio wishes to improve the reproducibility of the work that we publish. This form provides structure for consistency and transparency in reporting. For further information on Nature Portfolio policies, see our Editorial Policies and the Editorial Policy Checklist.

Statistics

For all statistical analyses, confirm that the following items are present in the figure legend, table legend, main text, or Methods section.

n/a	Confirmed	
☐	☒	The exact sample size (*n*) for each experimental group/condition, given as a discrete number and unit of measurement
☐	☒	A statement on whether measurements were taken from distinct samples or whether the same sample was measured repeatedly
☐	☒	The statistical test(s) used AND whether they are one- or two-sided<br>*Only common tests should be described solely by name; describe more complex techniques in the Methods section.*
☐	☒	A description of all covariates tested
☐	☒	A description of any assumptions or corrections, such as tests of normality and adjustment for multiple comparisons
☐	☒	A full description of the statistical parameters including central tendency (e.g. means) or other basic estimates (e.g. regression coefficient) AND variation (e.g. standard deviation) or associated estimates of uncertainty (e.g. confidence intervals)
☐	☒	For null hypothesis testing, the test statistic (e.g. *F*, *t*, *r*) with confidence intervals, effect sizes, degrees of freedom and *P* value noted<br>*Give P values as exact values whenever suitable.*
☒	☐	For Bayesian analysis, information on the choice of priors and Markov chain Monte Carlo settings
☐	☒	For hierarchical and complex designs, identification of the appropriate level for tests and full reporting of outcomes
☐	☒	Estimates of effect sizes (e.g. Cohen's *d*, Pearson's *r*), indicating how they were calculated

Our web collection on statistics for biologists contains articles on many of the points above.

Software and code

Policy information about availability of computer code

Data collection	[Amplicon sequencing data] After demultiplexing with sdm as part of the LotuS pipeline (v. 1.60) without allowing for mismatches, fastq raw amplicon sequencing files (2x250, Miseq illumina) were further analysed per sample using DADA2 pipeline (v. 1.6).<br>[Microbial load] The flow cytometry analysis was performed using a C6 Accuri flow cytometer (v.1.0.264.21, BD Biosciences).
Data analysis	[Amplicon sequencing data] QMP profiles were created using QMP R-script (https://github.com/raeslab/QMP-Microbiome-CRC-confounders) to rarefy the profiles to even sampling depth using the microbial load and R package: RasperGade16S (v. 0.0.1).<br>[Statistical analyses] Statistical analyses were performed on Rstudio with R(v.4.2.1) using the following R packages: phyloseq(v. 1.36.0), vegan (v. 2.6.2), coin(v. 1.4.2), effectsize(v. 0.8.3), vcd(1.4.11), DirichletMultinomial(v. 1.34.0), pairwiseAdonis(v. 0.4.1) and microbiome(v. 1.14.0).

For manuscripts utilizing custom algorithms or software that are central to the research but not yet described in published literature, software must be made available to editors and reviewers. We strongly encourage code deposition in a community repository (e.g. GitHub). See the Nature Portfolio guidelines for submitting code & software for further information.

Data

Policy information about availability of data

All manuscripts must include a data availability statement. This statement should provide the following information, where applicable:

- Accession codes, unique identifiers, or web links for publicly available datasets
- A description of any restrictions on data availability
- For clinical datasets or third party data, please ensure that the statement adheres to our policy

Raw amplicon sequencing data and metadata reported in this study have been deposited in European Nucleotide Archive with accession codes EGAS00001007413. FGFP 16S rRNA gene sequencing data and metadata are available at the European Genome-phenome Archive (EGAS00001003296). The diagnosis metadata and processed microbiome data required for the reanalysis are provided as Supplementary Table 1 and Supplementary Table 14, respectively.
Formatted Silva set 'SLV_nr99_v138.1' files were downloaded from: https://zenodo.org/records/4587955/files/silva_nr99_v138.1_wSpecies_train_set.fa.gz?download=1 (silva_nr99_v138.1_wSpecies_train_set.fa.gz) and https://zenodo.org/records/4587955/files/silva_species_assignment_v138.1.fa.gz?download=1 (silva_species_assignment_v138.1.fa.gz).
The 9 colorectal cancer cohort MetaPhlAn 3.0 profiles were collected from curatedMetagenomicData, study names: FengQ_2015, HanniganGD_2017, ThomasAM_2018a, ThomasAM_2018b, VogtmannE_2016, WirbelJ_2018, YachidaS_2019, YuJ_2015, ZellerG_2014 (DOI: 10.18129/B9.bioc.curatedMetagenomicData).

Human research participants

Policy information about studies involving human research participants and Sex and Gender in Research.

Reporting on sex and gender	Results only refer to sex. Gender was not recorded.
Population characteristics	A total of eight variables were found significantly associated (FDR < 5%) with diagnostic groups (namely: age, BMI, calprotectin, reported hours of sleep, previous cancer (including CRC), diabetes treatment and high blood pressure). Younger patients were more likely to exhibit no evidence of colonic lesions, whereas patients with lesions tended to be older. Additionally, patients with adenomas had a higher BMI compared to those without lesions, while patients without lesions have lower levels of intestinal calprotectin
Recruitment	Patients were recruited through the study nurse following a standardized procedure. Briefly, we invited patients scheduled for lower gastrointestinal endoscopy or abdominal surgery for CRC removal at the UZL. After explaining the research project and upon expressed interest, the informed consent was signed, and stool sample collection materials were provided. After colonic examination, patients were assigned to one of the diagnosis groups. In the case of the CLT group without colonic lesions, there might be an intrinsic increased risk of colorectal cancer due to the clinical necessity of a colonic examination.
Ethics oversight	Medical ethics committee of the UZL [Ethical approval number: S57084].

Note that full information on the approval of the study protocol must also be provided in the manuscript.

Field-specific reporting

Please select the one below that is the best fit for your research. If you are not sure, read the appropriate sections before making your selection.

☒ Life sciences ☐ Behavioural & social sciences ☐ Ecological, evolutionary & environmental sciences

For a reference copy of the document with all sections, see nature.com/documents/nr-reporting-summary-flat.pdf

Life sciences study design

All studies must disclose on these points even when the disclosure is negative.

Sample size	No sample size calculation was performed. Cohort sizes were determined based on previous findings (Falony et al., Science 2016).
Data exclusions	Taxa unclassified at the species level or present in <5 % of samples per each diagnosis group were excluded from the statistical analyses.
Replication	Several correlation on species abundance and BMI (2 of 6), fecal calprotectin (17 of 29), and moisture content values (29 of 50) in the LCPM were replicated in the FGFP cohort.
Randomization	Not applicable: this was a cross-sectional study, not a randomized study. No intervention was performed on subjects, and therefore no random allocation into groups.
Blinding	Not applicable: this was a cross-sectional study, not a randomized study. Investigators were not blinded during data collection and analyses.

Reporting for specific materials, systems and methods

We require information from authors about some types of materials, experimental systems and methods used in many studies. Here, indicate whether each material, system or method listed is relevant to your study. If you are not sure if a list item applies to your research, read the appropriate section before selecting a response.

Materials & experimental systems

n/a	Involved in the study
☒	☐ Antibodies
☒	☐ Eukaryotic cell lines
☒	☐ Palaeontology and archaeology
☒	☐ Animals and other organisms
☐	☒ Clinical data
☒	☐ Dual use research of concern

Methods

n/a	Involved in the study
☒	☐ ChIP-seq
☐	☒ Flow cytometry
☒	☐ MRI-based neuroimaging

Clinical data

Policy information about clinical studies

All manuscripts should comply with the ICMJE guidelines for publication of clinical research and a completed CONSORT checklist must be included with all submissions.

Clinical trial registration: The study protocol was registered at clinicaltrial.gov (NCT02947607)

Study protocol: The study protocol is available at https://clinicaltrials.gov/ct2/show/NCT02947607

Data collection: The final Leuven CRC Progression Microbiome (LCPM) study cohort consisted of 589 patients. The screening cohort included 650 volunteers referred for colonoscopy screening and colonic resections at UZL between 2017-2018, who provided a stool sample before the colonic procedure. Most participants were from the Flemish region of Belgium. These participants were classified within three diagnostic groups according to a thorough colonoscopy and clinical assessment: 1) patients without evidence of colonic lesions (CTL), 2) patients with polyps (n< 10 and size between 6 to 10 mm) (ADE), and 3) patients with CRC (CRC).

Outcomes: The hypotheses tested in this manuscript were not originally specified as part of the planned outcomes for the NCT02947607 study. The primary outcome of the project, characterization of "Differential host microbiome composition and abundance in healthy, adenoma, and CRC patients and its correlation to CRC risk features and host genomic and transcriptomic components," is not addressed in this manuscript.
While the secondary outcome, involving the analysis of "Host microbiome composition and abundance data generated from saliva, stool, and colonic biopsies using amplicon-based 16S ribosomal RNA sequencing," is partially explored in this manuscript, particularly regarding stool microbial communities, a comprehensive examination across all specified sample types and methodologies outlined in the study protocol, were not fully developed.

Flow Cytometry

Plots

Confirm that:

☒ The axis labels state the marker and fluorochrome used (e.g. CD4-FITC).

☒ The axis scales are clearly visible. Include numbers along axes only for bottom left plot of group (a 'group' is an analysis of identical markers).

☒ All plots are contour plots with outliers or pseudocolor plots.

☒ A numerical value for number of cells or percentage (with statistics) is provided.

Methodology

Sample preparation: 0.2 g frozen (–80 °C) aliquots were dissolved in physiological solution to a total volume of 100 ml (8.5 g l–1 NaCl; VWR International). Subsequently, the slurry was diluted 1,000 times. Samples were filtered using a sterile syringe filter (pore size of 5 µm; Sartorius Stedim Biotech). Next, 1 ml of the microbial cell suspension obtained was stained with 1 µl SYBR Green I (1:100 dilution in dimethylsulfoxide; shaded for 15 min of incubation at 37 °C; 10,000 concentrate, Thermo Fisher Scientific).

Instrument: C6 Accuri flow cytometer (BD Biosciences)

Software: BD Accuri CFlow software v.1.0.264.21 (BD Biosciences)

Cell population abundance: not applicable. No sorting of fractions was performed.

Gating strategy: Fluorescence events were monitored using the FL1 533/30 nm and FL3 > 670 nm optical detectors. In addition, forward and

Gating strategy

sideward scattered light was collected. The BD Accuri CFlow software was used to gate and separate the microbial fluorescence events on the FL1/FL3 density plot from background events. The gated fluorescence events were evaluated on the forward and sideward density plot, as to exclude remaining background events.

☒ Tick this box to confirm that a figure exemplifying the gating strategy is provided in the Supplementary Information.
